# High-Dose Irradiation Induces Cell Cycle Arrest, Apoptosis, and Developmental Defects during *Drosophila* Oogenesis

**DOI:** 10.1371/journal.pone.0089009

**Published:** 2014-02-13

**Authors:** Hee Jin Shim, Eun-Mi Lee, Long Duy Nguyen, Jaekyung Shim, Young-Han Song

**Affiliations:** 1 Ilsong Institute of Life Science, Hallym University, Anyang, Gyeonggi-do, Korea; 2 Department of Molecular Biology, Sejong University, Seoul, Korea; St. Georges University of London, United Kingdom

## Abstract

Ionizing radiation (IR) treatment induces a DNA damage response, including cell cycle arrest, DNA repair, and apoptosis in metazoan somatic cells. Because little has been reported in germline cells, we performed a temporal analysis of the DNA damage response utilizing *Drosophila* oogenesis as a model system. Oogenesis in the adult *Drosophila* female begins with the generation of 16-cell cyst by four mitotic divisions of a cystoblast derived from the germline stem cells. We found that high-dose irradiation induced S and G2 arrests in these mitotically dividing germline cells in a *grp/Chk1*- and *mnk/Chk2*-dependent manner. However, the upstream kinase *mei-41*, *Drosophila ATR* ortholog, was required for the S-phase checkpoint but not for the G2 arrest. As in somatic cells, *mnk/Chk2* and *dp53* were required for the major cell death observed in early oogenesis when oocyte selection and meiotic recombination occurs. Similar to the unscheduled DNA double-strand breaks (DSBs) generated from defective repair during meiotic recombination, IR-induced DSBs produced developmental defects affecting the spherical morphology of meiotic chromosomes and dorsal-ventral patterning. Moreover, various morphological abnormalities in the ovary were detected after irradiation. Most of the IR-induced defects observed in oogenesis were reversible and were restored between 24 and 96 h after irradiation. These defects in oogenesis severely reduced daily egg production and the hatch rate of the embryos of irradiated female. In summary, irradiated germline cells induced DSBs, cell cycle arrest, apoptosis, and developmental defects resulting in reduction of egg production and defective embryogenesis.

## Introduction

In response to DNA damage, normal metazoan cells activate a DNA damage response resulting in cell cycle arrest, repair of the damaged DNA, and/or apoptosis. Many genes involved in this response are well conserved from yeast to mammals, suggesting that the pathway is important for maintaining genomic stability. The discovery of the *dp53* gene in *Drosophila* by genome sequencing made *Drosophila* a more useful model organism for studying the DNA damage response [1]. Studies in flies with mutations in the *Drosophila* orthologs for ATM (*dATM*), ATR (*mei-41*), Chk1 (*grp*), Chk2 (*mnk*), and p53 (*dp53*) have revealed that *dATM*, *mnk*, and *dp53* are required for DNA damage-induced apoptosis, whereas only *mei-41* and *grp* play a major role in cell cycle arrest. Most of these studies were performed with mitotically dividing somatic cells in early embryogenesis or imaginal discs from third instar larvae. Although daily egg production has been shown to be reduced by the high-dose irradiation of adult females several decades ago [2] and irradiation has been used in *Drosophila* genetics to induce mutagenesis, the irradiation-induced cellular response at the molecular level is relatively unknown. Here, we used *Drosophila* oogenesis as a model system to investigate the DNA damage response in germline cells.

*Drosophila* females have a pair of ovaries containing approximately 18 ovarioles, each containing a germarium and developmentally ordered stage 2–14 egg chambers. *Drosophila* oogenesis begins at the anterior tip of the ovariole called the germarium, which contains three developmentally distinct regions. The germline stem cells in the germarium divide to produce a specialized germline cell called the “cystoblast”, which then performs four rounds of mitotic divisions with incomplete cytokinesis generating a 16-cell cyst in region 1. In region 2, all 16 cells perform a premeiotic S phase, and only one of them is determined as an oocyte and undergoes meiotic recombination. The other 15 cells become nurse cells and perform endocycles. The somatic stem cells located in region 2 proliferate to produce a monolayer of somatic follicle cells that surrounds the 16-cell germline cyst in region 3, which then buds off from the germarium and produces egg chambers moving posteriorly as they develop. Somatic follicle cells undergo mitotic divisions in stage 2–6 egg chambers and change from mitotic cell cycles to endoreduplication during stages 7–10. At later stages, the follicle cells cease genome-wide DNA replication and perform chorion gene amplification to produce the eggshell components. The *Drosophila* ovary, therefore, consists of germline and somatic cells performing various modes of cell division, including mitotic division, meiosis, endoreduplication, and gene amplification, depending on the developmental stages.

Proliferation, cell death, and development of the germline and somatic cells during *Drosophila* oogenesis are regulated by various internal and external factors. For example, poor nutrition reduces the rate of egg production by decreasing proliferation of the germline and somatic cells and inducing cell death at two precise developmental points: in region 2 within the germarium and in stage 7–10 egg chambers [3]. Additionally, DNA double-strand breaks (DSBs) are normally generated and repaired during meiotic recombination in the germarium. Unrepaired DSBs caused by mutations in repair-enzymes activate the *mei-41*- and *mnk*-dependent “meiotic checkpoint” resulting in developmental defects affecting the morphology of the oocyte nuclei and dorsal-ventral patterning [4]. Here, we found that DSBs generated by ionizing radiation (IR) also result in cell cycle arrest, cell death, and developmental defects in the germline and somatic cells of the ovary. These defects ultimately affected the number of eggs laid by the irradiated female and the embryogenesis of those eggs, whereas most of the surviving first instar larvae developed into pupae and adults without affecting the genomic stability.

## Materials and Methods

### Drosophila strains

All *Drosophila* strains were maintained at 25°C. *Canton S* or *w^1118^* flies were used as wild type controls. *Df(3L)W4* (which contained a deficiency that removed *mus304/ATRIP*), *mus101^D1^* (*mus101/TopBP1* mutant), *mus304^D1^*, and *p53^5A-1-4^* (*dp53* mutant) flies were obtained from the Bloomington Stock Center. The *mus304* mutant used in this study was *Df(3L)W4/mus304^D1^*. *mnk^6006^* (*mnk* mutant) and *mei-41^D3^* (*mei-41* mutant) flies were provided by Dr. Theurkauf [5], [6] and *grp^Z5170^* (*grp* mutant) and *grp^Z5170^ lok^30^* (*grp mnk* double mutant) flies were obtained from Dr. Sekelsky [7].

### Immunofluorescence staining of ovary

Four- to six-day-old females grown in the presence of yeast pastes for two days were mock treated or irradiated in a Cs^137^ γ-irradiator at 40 Gy. Immunofluorescence staining was performed using the standard procedure [8] with slight modifications. Briefly, the ovaries were dissected and fixed in a solution containing 100 µL of the buffer B fixative [1 vol of 37% formaldehyde (Sigma), 1 vol of buffer B (100 mM KH_2_PO_4_/K_2_HPO_4_ pH 6.8, 150 mM NaCl, 450 mM KCl, and 20 mM MgCl_2_·6H_2_O), and 4 vol of distilled water] and 600 µL of heptane for 5 min. The ovaries were washed three times for 5 min with PBS and PBT (0.1% Triton X-100 in PBS) and incubated in PBT for 1 h. Blocking was performed in PBT containing 5% normal goat serum (donkey serum for Vasa) for 1 h. The primary antibody diluted in blocking solution was added to the ovary and incubated overnight at 4°C. After washing with PBT three times for 5 min each, the ovary was incubated with the secondary antibody diluted in the blocking solution overnight at 4°C and washed with PBT twice for 10 min each. The DNA was stained with 1 µg/mL DAPI or 5 µg/mL propidium iodide.

To detect the cells in S phase, the ovaries were dissected in Grace's insect medium (WelGENE) and incubated in 50 µg/mL BrdU (Sigma) diluted in Grace's medium for 30 min at room temperature. The ovaries were washed three times in Grace's medium and fixed in the buffer B fixative solution for 20 min. The ovaries were washed three times for 10 min each in PBS and two times for 10 min each in 1× DNase buffer. The samples were treated with 0.05 U/µL DNase (Promega) for 1 h at 37°C and washed with PBT four times for 10 min each. The blocking and antibody staining were performed as above.

To detect the apoptotic cells, TUNEL staining was performed using the ApopTag® Red *In Situ* Apoptosis Detection Kit (Millipore). The dissected ovaries were agitated gently for 15 min in a solution containing 100 µL of the buffer B fixative and 600 µL of heptane. They were washed 3 times for 10 min each sequentially with PBS, PBT, and 1X BSS [54 mM NaCl, 40 mM KCl, 7.4 mM MgSO_4_, 2.4 mM CaCl_2_^.^2H_2_O, 4.8 mM Tricine, 0.36% (wt/vol) glucose, and 1.7% (wt/vol) sucrose]. The samples were treated with 10 µg/mL of proteinase K in PBS for 2 min and washed in PBT for 5 min twice. The ovaries were fixed in 2% paraformaldehyde for 10 min, washed for 5 min in PBT 4 times, and incubated in an equilibration buffer for 1 h at room temperature. The samples were incubated in a reaction mixture (reaction buffer and TdT in a 7∶3 ratio) for 3 h at 37°C in a humid chamber. The reaction was terminated by incubating in a stop reaction mix, which was generated by diluting the stop/wash buffer in dH_2_O (1∶34), for 30 min at 37°C. The ovaries were washed in PBT for 5 min 4 times and blocked in BSN (5% goat serum, and 0.3% triton X-100 in BSS) for 1 h. The samples were incubated with a Rhodamine conjugated anti-DIG antibody (1∶100 in BSN, Roche) at 4°C overnight. The ovaries were washed in PBT for 20 min 4 times and mounted in 0.5% n-propyl gallate dissolved in glycerol.

The primary antibodies used in this study include antibodies against phospho-histone H3 (Ser 10) (1∶300, Millipore), BrdU (1∶40, BD Biosciences), γH2Av (1∶400, Rockland), Orb (1∶1, a mixture of clone 4H8 and 6H4, Developmental Studies Hybridoma Bank), and Vasa (1∶20, Santa Cruz). The secondary antibodies were purchased from Jackson ImmunoResearch and Molecular Probes. The ovaries were visualized using a confocal laser scanning microscope (Carl Zeiss, LSM 700) or a fluorescence microscope (Olympus, IX71).

### Developmental phenotypes of the embryos derived from irradiated females

To determine the fecundity and hatch rate of the embryos derived from irradiated females, two- or three-day-old wild type virgin females were irradiated with 0 or 40 Gy and put into an egg laying cage with the same number of males. The eggs were collected every 24 h, and the number of eggs and the larvae hatched from the eggs were counted. Day 1 corresponds to the egg collection during the first 24 h after irradiation. The hatch rate was calculated by the number of larvae generated from the eggs in each collection. The bristle defects were examined as previously described [9].

## Results

### Cell cycle arrest of germline and somatic cells after irradiation

**Figure 1:**
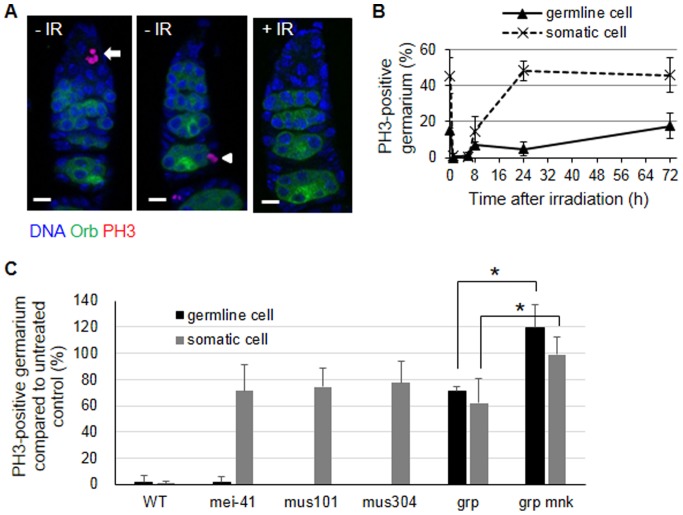
IR-induced G2 arrest in mitotically dividing germline and somatic cells in wild type and checkpoint mutant ovaries. The wild type or indicated mutant adult females were irradiated with 0 or 40 Gy and the ovaries were dissected at the indicated time points. The ovaries were stained with an anti-Orb (green) and anti-phospho-histone H3 (PH3) antibody (red) to detect germline cells in region 2/3 and mitotic cells, respectively. The DNA was stained with DAPI (blue). (A) Representative images of the wild type germarium containing PH3-stained germline cells (arrow) or somatic follicle cells (arrowhead) are shown in the absence of irradiation (-IR) or 1 h after irradiation (+IR). Germariums are oriented as the anterior to the top. Bar, 5 µm. (B) The wild type ovaries were dissected 1, 6, 8, 24, and 72 h after 40 Gy of irradiation and stained with PH3. The number of germariums containing PH3-positive germline cells in region 1 and somatic cells in region 3 were counted. The values represent the percentage of germarium containing PH3-positive germline cells (solid line) and somatic cells (dotted line). The data are presented as the mean and standard deviation of three independent experiments. The total number of germarium for each time point was at least 150. (C) The ovaries of checkpoint mutant adult females were stained with PH3 1 h after 40 Gy. The germariums containing PH3-stained germline cells (black bar) or somatic cells (gray bar) were counted. The values represent the relative percentage of PH3-positive germariums in irradiated ovaries compared to the untreated controls. The data are presented as the mean and standard deviation of three independent experiments. The total number of germariums for each genotype was at least 150. * *p*<0.05 (the *grp* mutant versus the *grp mnk* double mutant).

To understand the cellular response induced by high-dose irradiation in the germline cells, adult female flies were irradiated at 40 Gy. Although 40 Gy irradiation is the LD_50_ for larvae [10], this dose did not affect the viability of adult females during the course of our experiment. After irradiation, cell cycle arrest was determined by staining the ovary with an antibody against phospho-histone H3 (PH3) to detect the mitotic cells. In the absence of irradiation, PH3 staining was observed in the germline-derived 2-, 4-, and 8-cell-cysts that were dividing synchronously in region 1 and the somatic follicle cells undergoing asynchronous division in region 3 (Figure 1A, left two panels). In the absence of irradiation, the percentage of germarium containing PH3-positive germline cells in region 1 and somatic cells in region 3 were 14.9±1.8% and 45.6±9.8%, respectively (Figure 1B). At 1 h after irradiation, PH3-positive cells were not detected in either the germline or the somatic cells (Figure 1A, right panel and 1B). This result suggests that both germline and somatic cells arrest at G2 phase, assuming that the length of G2 phase of these cells is less than 1 h. At 8 h post-irradiation, the percentage of PH3-positive germarium increased in both cell types and was restored to the untreated level by 72 h in the germline cells and 24 h in the somatic cells (Figure 1B). These results showed that the mitotically dividing germline cells arrest at G2 after irradiation and resume the cell cycle slightly slower than the somatic cells.

In larval somatic cells, *Drosophila* orthologs of *ATR (mei-41)*, *TopBP1 (mus101)*, *ATRIP (mus304)*, *Chk1 (grp)*, and *Chk2 (mnk, also known as lok)* genes are required for IR-induced G2 arrest [1], [11], and we tested whether these genes also function in the germline and somatic cells of the ovary. The irradiated *grp* mutant contained 70.8±4.1% and 62.3±18.1% PH3-positive germarium in region 1 and 3, respectively, compared to the untreated control, and these relative percentages were significantly increased in the irradiated *grp mnk* double mutant in both the germline (119.6±17.8%) and the somatic cells (99.0±13.3%) (Figure 1C). This is similar to previous results in larval imaginal discs [12], suggesting that *grp* and *mnk* cooperate to induce G2 arrest in mitotically dividing germline and somatic cells in the ovary. However, females mutant for the upstream kinase of *grp* and its mediators, *mei-41*, *mus101*, and *mus304* were able to induce G2 arrest upon IR treatment in the germline cells even though they were defective in the somatic follicle cells. These results suggest that a distinct sensor kinase(s) other than Mei-41 may be utilized in germline cells for IR-induced G2 arrest, but the signal still converges on the same effector kinases, Grp and Mnk.

**Figure 2:**
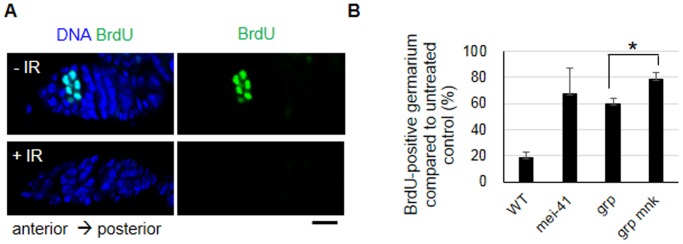
IR-induced S-phase checkpoint in mitotically dividing germline cells in wild type and checkpoint mutant females. The BrdU incorporation assay was performed in wild type and checkpoint mutant ovaries 1(A) Representative images of a wild type germarium assayed for BrdU incorporation (green) in the germline cells before and after irradiation are shown. The DNA was stained with DAPI (blue). Bar, 10 µm. (B) The number of germariums containing a strong BrdU signal in region 1 were counted. The values represent the relative percentage of germarium containing BrdU-positive germline cells in irradiated samples compared to the untreated controls. The data are presented as the means and standard deviations of three independent experiments. The total number of germarium for each cell type at different time points were at least 220. * *p* = 0.0016 (the *grp* mutant versus the *grp mnk* double mutant).

In addition to G2 arrest, larval somatic cells activate the S-phase checkpoint by slowing down DNA replication in response to IR in a *mei-41*- and *grp*-dependent way [13]. We performed a BrdU incorporation assay in the wild type females and found that 54.1±7.5% of the germarium contained synchronously dividing germline cells in region 1; 43.4±11.9% had strong BrdU signal (Figure 2A), and 15.7±9.4% were weakly stained (data not shown). The percentage of the irradiated germarium containing germline cells with a strong BrdU signal decreased to 18.9±3.9% of the untreated control (from 43.4±11.9% to 7.9±1.0%; Figure 2B). Similar to the larval somatic cells, the percentage of S-phase germline cells in the irradiated samples remained as high as 67.2±19.9% and 59.6±4.2% of the untreated control in the *mei-41* and *grp* mutant females, respectively. The percentage of S-phase cells in the *grp* mutant further increased to 78.3±5.5% in the *grp mnk* double mutant, suggesting that *grp* and *mnk* also cooperate for the IR-induced S-phase checkpoint in mitotically dividing female germline cells.

### Apoptosis in the germline cells after irradiation

**Figure 3:**
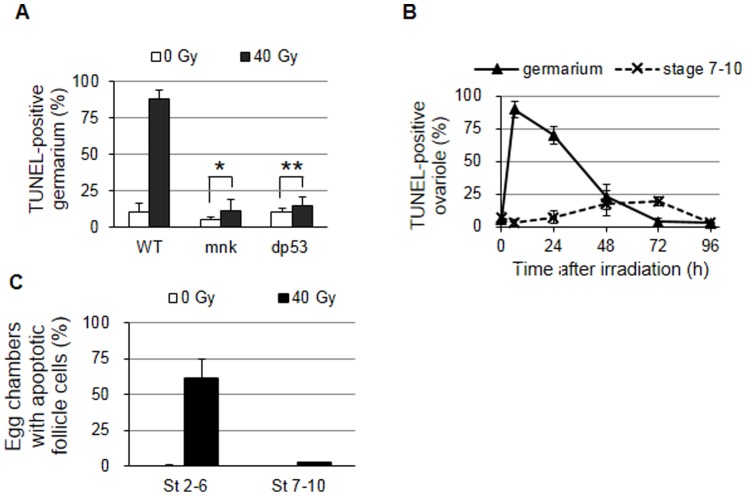
IR-induced apoptosis during oogenesis. The wild type or mutant adult females were irradiated at 40 Gy, and the ovaries were stained by using the TUNEL method to detect the apoptotic cells. (A) The values represent the percentage of wild type or the indicated mutant ovarioles containing TUNEL-positive germline cells in region 2 at 6 h after irradiation. The data are presented as the mean and standard deviation of two independent experiments. The total number of ovarioles counted for each genotype was at least 100. * *p* = 0.085. ** *p* = 0.21. (B) The values represent the percentage of wild type ovarioles containing TUNEL-positive germline cells in region 2 (solid line) or degenerating egg chambers at stages 7-10 (dotted line) at 0, 6, 24, 48, 72, and 96 h after irradiation. The data are presented as the mean and standard deviation of two independent experiments. The total number of ovarioles counted at each time point was at least 70. (C) The values represent the percentage of wild type egg chambers at stages 2–6 or 7–10 containing more than 5 TUNEL-positive follicle cells 6 h after irradiation. The total number of egg chambers counted at each time point was at least 150.

*Drosophila* oogenesis is sensitive to environmental stresses such as poor nutrition which induces apoptosis at two distinct developmental stages: in the germarium region 2 and the stage 7–10 egg chambers [3]. We tested whether IR also induced apoptosis and found that 6 h after 40 Gy irradiation 89.7±6.3% of the germarium contained TUNEL-positive cysts in region 2 (Figure 3A). This apoptosis occurred through *mnk* and *dp53* because apoptosis was not significantly increased in the *mnk* and the *dp53* mutants. When the time course analysis was performed in wild type females, apoptosis in the germarium remained high (70.0±6.6%) 24 h after IR but gradually decreased to lower level (22.8±9.7%) at 48 h (Figure 3B). Interestingly, cell death in the egg chambers at stage 7–10 was not significantly increased during the first 24 h after irradiation, although approximately 20% of the ovarioles contained degenerating egg chambers at 48 and 72 h. In addition to the apoptosis observed in the germline cells, stage-specific TUNEL staining was also observed after irradiation in the somatic follicle cells that encircle the 16 germline cells. When egg chambers containing more than 5 TUNEL-positive cells were counted as apoptotic, cell death in the follicles cells at stages 2–10 was not detected in the absence of irradiation (Figure 3C). At 6 h after irradiation, the percentage of egg chambers containing apoptotic follicle cells undergoing mitotic divisions at stage 2–6 increased to 61%. Most of the somatic follicle cells undergoing endocycles at stage 7–10 were not TUNEL positive, as previously shown for endocycling nurse cells [14].

### Meiotic checkpoint after irradiation

**Figure 4:**
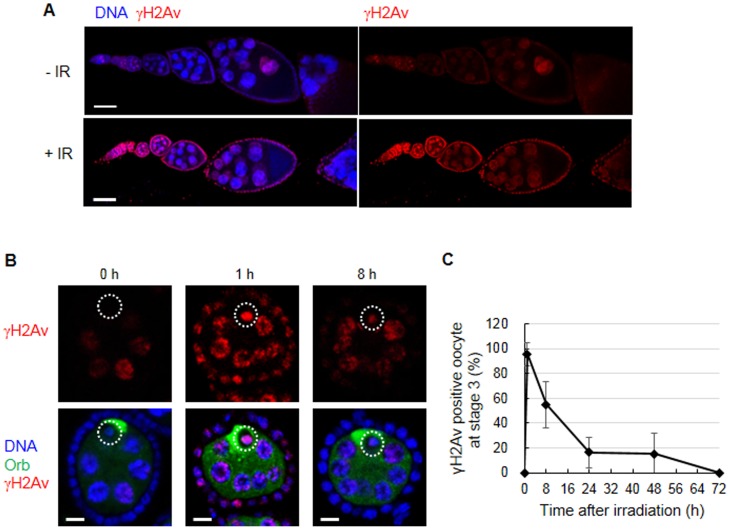
Generation and repair of DSBs detected by H2Av phosphorylation in oocytes after IR treatment. The wild type females were irradiated at 40 Gy, and the ovaries were stained for γH2Av (red), DNA (blue), and Orb (green). (A) Representative confocal images of ovariole before and 1 h after irradiation are shown. Bar, 50 µm. (B) Representative confocal images of stage 3 egg chambers 0, 1, and 8 h after irradiation are shown. The oocytes containing a high level of Orb proteins are indicated by a white circle. Bar, 5 µm. (C) The percentage of oocytes at stage 3 stained with γH2Av was determined at 0, 1, 8, 24, 48, and 72 h after irradiation. The data are presented as the mean and standard deviation of two independent experiments. The total number of oocytes at each time point was at least 14.

During meiotic recombination in normal oogenesis, DSBs are generated and repaired in the oocyte in region 2 of the germarium. Mutant females that are defective in the repair of these DSBs activate the *mnk*-dependent meiotic checkpoint resulting in developmental defects [4]. To test whether IR generates DSBs and induces the meiotic checkpoint, we first tested DSBs production. In mammals, DSBs can be visualized by the γH2AX foci, which is generated by the rapid phosphorylation of serine at the carboxyl terminus of the histone variant H2AX at the site of the breaks [15]. The ovaries were stained with an antibody that specifically recognizes the phosphorylated form of H2Av (γH2Av), the *Drosophila* ortholog of H2AX. In the absence of irradiation, γH2Av signal was detected in the germarium region 2 and endocycling nurse cells and somatic follicle cells where the DSBs are known to be generated during normal development ([14], [16], [17] and Figure 4A). The intensity of γH2Av signal was significantly increased in both mitotic cycling and endocycling cells 1 h after irradiation as previously shown ([14] and Figure 4A). We analyzed the DSB formation in the oocyte at stage 3, because most of the H2Av is removed from the nucleosome of the germline cells at stages 4–5 during oogenesis [18]. Most of the oocytes at stage 3 (96.4±9.4%) showed a strong γH2Av signal 1 h after irradiation (Figure 4B). The percentage of oocytes showing a γH2Av signal was decreased to 55.0±18.4% and 16.3±12.0% at 8 and 24 h after irradiation, and the signal was undetectable at 72 h (Figure 4C). At 8 h after irradiation, the intensity of the γH2Av signal was also decreased to approximately 30% of that at 1 h post irradiation. This result suggests that the signaling event leading to H2Av phosphorylation or the DSBs themselves generated by IR reach a maximum at 1 h and are rapidly restored in the oocyte at stage 3.

**Figure 5:**
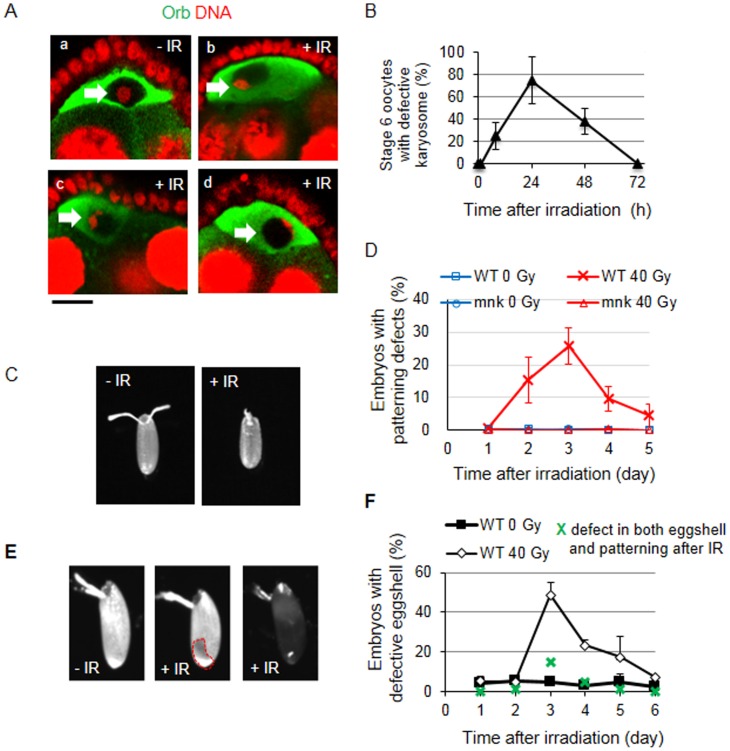
Meiotic checkpoint and defective eggshell induced by IR treatment. (A–B) Karyosome defects after IR treatment. Wild type females were irradiated at 40 Gy, and the ovaries were stained for DNA with propidium iodide (red) and for oocyte cytoplasm with an anti-Orb antibody (green). (A) Representative confocal images of stage 6 egg chambers before (a) and after irradiation (b, c, and d) are shown. Bar, 10 µm. The nuclei of oocytes are indicated with a white arrow. (B) The percentage of oocytes at stage 6 with defective karyosome was counted at 0, 1, 8, 24, 48, and 72 h after irradiation. The morphology of the karyosome similar to (Ad) was considered as defective. The data are presented as the mean and standard deviation of at least two independent experiments. The total number of oocytes at each time point was at least 21. (C–D) Dorsal-ventral patterning defects of the embryos laid by the irradiated females. (C) Representative images of a normal embryo from an untreated female (-IR) and a defective embryo from a wild type female irradiated at 40 Gy (+IR) are shown. (D) Wild type or *mnk* mutant females were irradiated at 40 Gy and the embryos were collected every 24 h. The percentage of embryos with dorsal-ventral patterning defects were counted each day. Day 1 corresponds to the embryos collected 0–24 h after irradiation. The data are presented as the mean and standard deviation of at least two independent experiments. At least 100 embryos were observed for each time point in each experiment. (E–F) Eggshell defects in the embryos derived from irradiated females. (E) The embryos derived from the untreated (-IR, normal eggshell) and 40 Gy irradiated (+IR) females are shown. The embryos from irradiated female exhibited irregular eggshells (second panel, indicated in the red dotted line) or lucid eggshells (third panel). (F) The embryos were collected from the irradiated females every 24 h, and the percentages of the embryos with thin eggshell phenotypes were counted. Day 1 represents the embryos collected at the first 24 h after irradiation. The data are presented as the mean and standard deviation of three independent experiments. At least 100 embryos were observed for each time point.

We tested whether the DSBs induced by irradiation also induced the meiotic checkpoint. After completing meiotic recombination, the chromosomes of the oocytes cluster together by stage 3 to form a spherical body called the karyosome, which is deformed in the presence of the unrepaired DSBs generated during meiotic recombination. We observed the oocytes at stages 3 and 6 to determine whether irradiation induces defects in karyosome morphology. In the absence of irradiation, the karyosome of all the oocytes observed at stages 3 (n = 24) and 6 (n = 21) appeared spherical (Figure 5Aa). After irradiation, various changes in karyosome morphology were detected. The irradiated karyosome exhibited ellipsoidal (Figure 5Ab), irregular (Figure 5Ac), or threadlike structures (Figure 5Ad) that appeared attached to the nuclear membrane delineated by the cytoplasmic Orb staining. Since the threadlike karyosome can be clearly distinguished from untreated control, we counted the oocyte with this phenotype as defective. The karyosome defects in the oocytes at stage 6 reached a maximum (75.0±21.5%) at 24 h and slowly reduced to basal level by 72 h (Figure 5B). By contrast, the oocytes at stage 3 did not show karyosome defects after irradiation. Assuming that the progression of the oogenesis after irradiation occurs at the same speed as previously reported [19], stage 6 egg chambers were at stage 3 at the time of irradiation when the maximum karyosome defect was observed.

We also tested whether the *mnk*-dependent dorsal-ventral patterning defect was induced in the eggs collected each day after irradiation. In the absence of irradiation, the dorsal-ventral patterning reflected as the presence of the two prominent anterior respiratory structures, the dorsal appendages, of the embryos from both the wild type and the *mnk* mutant females were normal. Defects in the dorsal appendages of the embryos laid by the irradiated females increased up to 25.6±5.5% in the embryos collected until 3 days after irradiation (Figure 5C and 5D). The embryos derived from the irradiated *mnk* mutant females did not show these defects, suggesting that the meiotic checkpoint induced by the exogenously generated DNA damage occurred through the same signaling pathway as that is induced by endogenously generated DSBs.

During the course of the experiment we observed that some embryos from the irradiated females had a thin eggshell. The chorion genes encode the major protein components of the eggshell and a thin eggshell phenotype is observed if this developmentally regulated gene amplification does not occur. In the absence of irradiation, the percentage of eggs with a thin eggshell was very low (2.1%–5.1%, Figure 5F). The eggs laid by the irradiated females exhibited eggshells that were irregular or far more translucent than the normally opaque wild type chorion indicative of a thin eggshell (Figure 5E, second or third panel). The percentage of eggs with a thin chorion was as high as 48.7±6.3% of the eggs collected for 24 h period between day 2 and 3 after irradiation. Among these, 30.2% had an irregular eggshell and 18.5% were translucent. Only 14.9% (indicated by a green x in Figure 5F) had defects in both the eggshell and dorsal-ventral patterning, suggesting that these phenotypes occur through distinct signaling pathways. To test whether the thin eggshells were actually induced by reduced chorion gene replication, a BrdU incorporation assay was performed. The BrdU staining of follicle cells at stages 10–13 was not significantly reduced 1 h after irradiation (data not shown). The maximum defects in the eggshell was observed 3 days after irradiation, which is significantly longer than the time (∼10 h) between stages 10 and 14 during normal oogenesis [19]. Therefore, it is likely that the thin eggshell phenotype may not be caused by reduction of chorion gene amplification.

### Additional defects of the ovary observed in irradiated females

**Figure 6:**
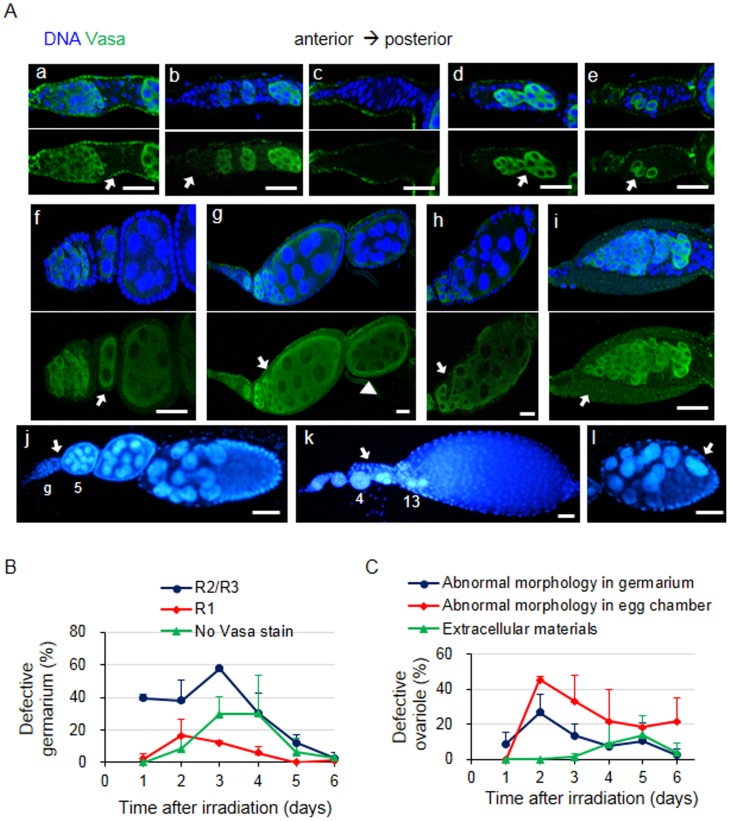
Morphological defects of ovarioles induced by high-dose irradiation. The wild type females were irradiated at 40 Gy, and the germline cells were stained with an anti-Vasa antibody (green) and DAPI (blue) each day after irradiation. (A) Various defects (indicated by an arrow) were observed in oogenesis after irradiation. Representative images exhibiting the following defects are shown: germarium lacking anti-Vasa-stained germline cells in region 3 and/or region 2b (a), in region 1 (b), or in the entire germarium (c); ovarioles lacking a couple of early stage egg chambers (j) and several late stage egg chambers (k); germarium exhibiting fusion of two cysts (d); fused egg chambers (g); single germline cells in the germarium (e); abnormal number of germline cells in the egg chambers (f); ovarioles containing smaller egg chambers at more posterior positions (g, arrowhead); egg chambers with an abnormal shape (h); and egg chambers containing mislocalized nurse cells (l). The germarium (g) and the stage of the egg chambers are shown in (j) and (k). The scale bars represent 20 µm (a–i) and 50 µm (j–l). (B) Irradiation induced loss of germline cells in the germarium. The percentage of germarium lacking anti-Vasa-stained germline cells in region 3 and/or 2 (blue dots), region 1 (red diamonds), or the entire germarium (green triangles) was counted each day after irradiation. The values represent the means and standard deviations from two independent experiments. At least 55 ovarioles were counted in total for each time point. (C) Morphological defects observed in the germarium and egg chambers. The percentage of germarium containing morphological defects in the germline cells (blue dots) or extracellular materials (green triangles) was counted. The percentage of ovarioles containing an abnormal egg chamber morphology (red diamonds) was also calculated. The values represent the means and standard deviations from two independent experiments. At least 55 ovarioles were counted in total for each time point.

The *Drosophila* ovariole contains a germarium and 6–7 progressively developing egg chambers with sequentially larger sizes. To test whether defects other than the ones we described above might have been induced during oogenesis by high-dose irradiation, the ovaries were stained with an anti-Vasa antibody and DAPI to stain germline cells and nuclei, respectively. Staining pattern of Vasa proteins and nuclei revealed the developmental or morphological changes that occurred in the germline cells by irradiation. At day 1, the defects were found only in the germarium (44.9±2.5% of the ovariole), and most of these defective germarium (39.5±2.4% of the ovariole) lacked germline cells in region 3 and/or part of region 2 (Figure 6Aa and 6B). This result reflects massive cell death in region 2 and also suggests that the progression of oogenesis occurred after irradiation. Starting from day 2, the ovarioles with less than 6–7 egg chambers, lacking successively later stages of the egg chambers over time, were observed. Most of these short ovarioles lacked a couple of early stage egg chambers at days 2 and 3 (Figure 6Aj), and ovarioles lacking several mid/late stage egg chambers increased at day 5 (Figure 6Ak). This further demonstrates the delayed cell death during mid-oogenesis and the progression of oogenesis. Additionally, we found that the frequency of germarium lacking germline cells in region 1 (Figure 6Ab) and the entire germarium (Figure 6Ac) increased at day 2 and 3, respectively (Figure 6B). This result suggests that the germline stem cells died after irradiation, subsequently producing the germarium lacking germline cells. Therefore, the time course analysis of ovarioles lacking germline cells supports the cell death we described above and also suggests the cell death of germline stem cells.

In addition to the ovarioles lacking germline cysts or egg chambers, we were able to detect various morphological defects in the cyst or egg chamber formation. The percentage of ovarioles containing morphological defects in the germarium and the egg chambers was very low at day 1, reached a maximum (26.8±10.3% and 45.4±2.1%, respectively) at day 2, and gradually decreased over time (Figure 6C). The defects included fusion of the cysts (Figure 6Ad) or egg chambers (Figure 6Ag, arrow), cysts containing single germline cells in the germarium (Figure 6Ae), egg chambers containing an abnormal number of germline cells (Figure 6Af, arrow), ovarioles containing smaller egg chambers at more posterior position (Figure 6Ag, arrowhead), and egg chambers with abnormal shape (Figure 6Ah, arrow) or mislocalized polyploid nurse cells (Figure 6Al, arrow). Additionally, germariums containing thick extracellular materials appeared during days 4–6 (Figure 6Ai and 6C). Starting at day 5, the ovarioles containing germarium and early stages of the egg chambers with a normal morphology increased, suggesting that normal oogenesis resumes in the surviving germline stem cells. These results show that various developmental defects were induced by high-dose irradiation, resulting in cyst and egg chambers with morphological defects that might have been removed by delayed apoptosis in mid-oogenesis.

### Number and development of embryos derived from irradiated females

**Figure 7:**
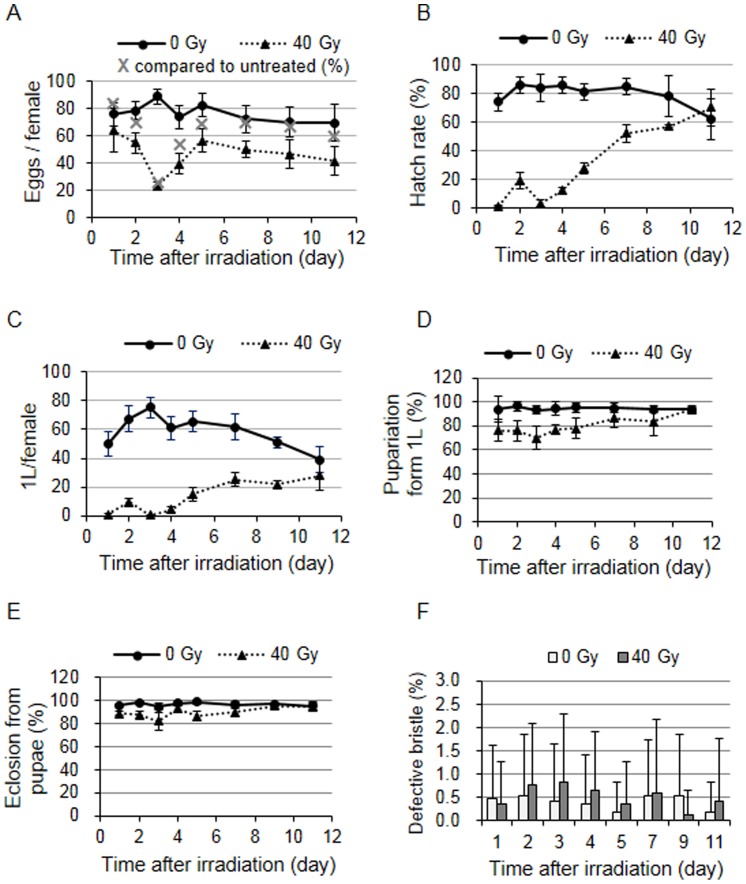
Fecundity, fertility, and the development of embryos derived from irradiated adult females. (A–C) Fecundity and fertility of the wild type irradiated females. Two- to three-day-old adult females were irradiated with 0 or 40 Gy and the embryos were collected every 24 h. Day 1 corresponds to the egg collection between 0 and 24 h. The number of embryos per female (A) and the hatch rate of these embryos (B) were determined. Two independent experiments were performed using at least 5 cages for each treatment in the experiment, and the cage contained 3 pairs of females and males. The data are presented as the mean and standard deviation of the number of embryos per female and the hatch rate of those embryos was calculated from each cage. The grey X in (A) represents the percentage of egg production by irradiated females compared to untreated females. (C) The number of hatched larvae derived from one female was calculated and shown as above. (D–F) Development of the first instar larvae produced from irradiated females. (D–E) First instar larvae (n = 100) were obtained from the daily collection of eggs laid by the females treated with 0 or 40 Gy, and the number of pupae and eclosed adults was counted. The values represent the percentage of the total number of pupae formed (D) and the percentage of total number of adults generated from the pupae (E). The data are presented as the mean and standard deviation of three independent experiments. (F) The *Minute* phenotypes were observed in the adult females produced from females treated with 0 or 40 Gy. The percentage of adults with defective bristles is shown. The data are presented as the mean and standard deviation of a total of 42 females observed in three independent experiments.

To understand the biological consequence of the phenomenon we described above, we determined the mean daily egg production and development of embryos derived from the irradiated females. Since cell lineage and histological studies showed that approximately 9 to 11 days are required for a stem cell to produce a mature egg [20], we examined the embryos for 11 days after irradiation. The embryos were collected for 24 h each day after irradiation and the number of embryos per female was calculated; Day 1 corresponds to the egg collection between 0 and 24 h. In the absence of irradiation, the number of eggs laid by wild type females was not significantly different until day 11, with an average of 76.3±6.7. During the first 24 h after irradiation (at day 1), the number of eggs laid by the irradiated females was not significantly different from the untreated females (63.8±16.6 vs. 75.9±8.5; *p* = 0.094). Then, the number of eggs gradually decreased to a minimum of 23.0±2.9 at day 3 (26% of the untreated control), reflecting massive cell death of the germline cells, and the number slowly recovered up to 56.4±8.3 at day 5. The number of eggs was approximately two-thirds that of the untreated females and remained that way until day 11 (Figure 7A, the grey x without a line). We followed the development of the embryos including the first instar larvae, pupae, and adult stages. In the absence of irradiation, the hatch rate of the eggs was not significantly different up to day 9, ranging between 74.1±6.0% and 86.3±5.6% (Figure 7B). Irradiation of the female severely reduced the hatch rate of the embryos to 1.3±0.7% and 2.9±2.4% at day 1 and 3, respectively, while it was 19.3±5.7% at day 2. This result suggests that sensitivity of the oocytes varies depending on the developmental stages when irradiated. The hatch rate gradually increased to that of untreated control by day 11. The actual number of first instar larvae produced by a single female was less than 15 during the first 4 days after irradiation and gradually increased to a level similar to the untreated control by day 11 (Figure 7C). We investigated whether development of the first instar larvae produced from irradiated females was affected and found that the percentage of pupariation and eclosion was slightly lower than that of the untreated control between days 2–5 and 1–7 after irradiation, respectively (Figure 7D and 7E, *p*<0.05). Additionally, we tested whether the adult progeny produced from the irradiated females maintains the genomic stability. When we assayed for the *Minute* phenotype, which is haploinsufficient for the development of large bristles, the percentage of defective bristles was less than 1% throughout the investigation period and did not seem to be affected by the irradiation of the female (Figure 7F). These results suggest that the defective oocytes generated by irradiation were eliminated mostly during oogenesis and embryogenesis, and most of the surviving first instar larvae developed to adult and were able to maintain genomic stability.

## Discussion

Normal metazoan cells elicit a DNA damage response, which is relatively uncharacterized in the germline cells, whose genomic stability is critical to maintaining the survival of the species. Here, we have investigated a DNA damage response in the *Drosophila* ovary containing germarium and progressively developing egg chambers of stages 2–14. We have found that high-dose irradiation induces cell cycle arrest, apoptosis, and developmental defects depending on the developmental stages of oogenesis. Cell cycle arrest in the mitotically dividing germline cells during early oogenesis occurs at the S and G2 phases. Grp is utilized as a major effector kinase, and Mnk has minor contribution, as in somatic cells. On the other hand, the upstream kinase Mei-41 is required for the IR-induced S-phase checkpoint but not for G2 arrest. Apoptotic cell death during early-oogenesis is induced rapidly in an *mnk*- and *dp53*-dependent way, whereas apoptosis in mid-oogenesis occurs with a delay. The developmental defects induced by irradiation affect the nuclear morphology of the oocytes, the dorsal-ventral patterning established during mid-oogenesis, and various morphological defects in cyst and egg chamber formation. These changes in oogenesis ultimately result in the reduction of egg production and the hatch rate of embryos derived from irradiated females. However, development of the surviving first instar larvae into pupae and adult stages were only slightly affected. These adults were without obvious morphological defects or genomic instability.

In the somatic cells of the *Drosophila* embryo and larval imaginal discs, DNA damage induces cell cycle checkpoints at S, G2/M, and the metaphase-anaphase transition [1]. We found that *grp* and *mnk* cooperate to induce the S and G2 checkpoints in mitotically dividing germline cells, as in larval somatic cells. Interestingly, mutants for *mei-41*, the upstream kinase of Grp and Mnk, were normal for the G2 arrest but defective in the S-phase checkpoint. This result is rather unexpected because the *mei-41* mutants showed severe defects in the IR-induced G2 arrest in most of the *Drosophila* cells tested. *Drosophila mei-41* belongs to a phosphatidylinositol 3-kinase-related kinase family including *dATM* and *SMG-1*. The role of *dATM* in IR-induced G2 arrest in larval imaginal discs has been described in response to low-dose irradiation [21] or the initiation of G2 arrest in response to high-dose irradiation [22]. Although the significance of *dATM* in IR-induced G2 arrest is not as prominent as *mei-41* in the soma, its contribution in the germline cells might have been enhanced. This possibility is supported by our previous result showing a high expression level of the *dATM* transcript in adult females compared to adult males or other developmental stages [22]. Moreover, the DNA damage response has been shown to be activated in the germline but not in the somatic cells of *C. elegans* due to the selective expression of ATM in the germline cells [23]. Alternatively, *dATM* and *mei-41* may function redundantly for G2 arrest in the germline cells.

In mammals, IR induces phosphorylation of the histone variant H2AX within several minutes, reaching a maximum level of phosphorylation approximately 30 min after irradiation [15]. Phosphorylation of H2Av, the *Drosophila* ortholog of mammalian H2AX, is detected in germline cells at sites of DSBs generated endogenously during meiotic recombination and exogenously by irradiation [16]. Time course analysis of γH2Av formation after irradiation has shown that this occurs more slowly in the early pachytene pro-oocytes in germarium region 2, where DSBs are generated and repaired during meiotic recombination, than in the oocytes at the later stages of pachytene in the germarium lacking endogenously generated DSBs [16]. We tested for γH2Av formation in the oocytes at stage 3 and found that the maximum response occurs at 1 h, at least 5 times faster than in the oocytes in the early pachytene. This result supports the hypothesis by Mehrotra and McKim that oocytes capable of producing developmentally regulated DSBs induce a delayed γH2Av response to both exogenous and endogenous DSBs or alternatively only to exogenous DSBs [16].

Two major developmental defects are induced by activation of the *mnk*-dependent meiotic checkpoint in response to unscheduled DSBs, which are produced by defects in DNA repair during meiotic recombination or in processing of repeat-associated siRNA that suppress germline retrotransposition [4], [24]. First, a dorsal-ventral patterning defect is induced by the Mnk-dependent phosphorylation of Vasa, an eIF4A-like translation initiation factor, and subsequent inhibition of the translation of *gurken*, a transforming growth factor-α-like signaling molecule [25]. In addition, defects in karyosome morphology occur through phosphorylation and inhibition of NHK1 by Mnk, resulting in a reduction of the phosphorylation of BAF1, a linker between the nuclear envelope and chromatin [26], [27]. We found that the DSBs generated by irradiation of adult females also induced the *mnk*-dependent meiotic checkpoint. Temporally, the condensation of oocyte chromosomes into a karyosome occurs by stage 3 and persists until stage 13, and the dorsal-ventral axis formation occurs in mid-oogenesis. Therefore, DSBs seem to activate Mnk in mid-oogenesis resulting in developmental defects rather than cell cycle arrest or apoptosis that occurred in early oogenesis. Because these cellular responses are directed by distinct targets of Mnk, substrate availability or prerequisite modification of the target protein may be responsible for the differential biological responses of Mnk activation during oogenesis.

The changes in the egg production of the irradiated females seem to occur in three phases. First, rapid reduction occurs during the first three days after irradiation, which can be explained by the cell cycle arrest of mitotically dividing cystoblasts in germarium region 1 and degeneration of germline cells in germarium region 2 and egg chambers at stages 7–10. The second phase during days 4–5 corresponds to the recovery, reflecting the resumption of cell cycle and cessation of cell death. During the last phase, 5–11 days after irradiation, the number of eggs produced by irradiated females is maintained at approximately 70% of the untreated controls. The 70% reduction of fecundity at the later time point may be explained by the death of germline stem cells, resulting in the decrease of the total number of stem cells in the females. The appearance of a germarium lacking germline cells in region 1, followed by an entire germarium and empty ovariole support this notion. Further analysis will be required to prove the death of germline stem cells after irradiation.

In addition to the reduced fecundity, the hatch rate of the laid embryos by irradiated females was severely reduced to 2.9% at day 3 and gradually increased to the control level by day 11. The reduction of the hatch rate can be explained by the three phenotypes we observed. First, the dorsal-ventral patterning defects of the eggs established during mid-oogenesis were maximally affected in the eggs laid at day 3 after irradiation. Second, the percentage of embryos with thin eggshells was maximum at day 3. The underlying mechanism of thin eggshell is not clear and does not seem to occur by the reduction of the chorion gene amplification. In mitotically dividing somatic cells, DNA damage induces intra-S phase checkpoint by inhibiting progression through S phase and initiation of later origins of replication, which are usually considered to maintain genomic stability by allowing time for DNA repair. The somatic follicle cells at stages 10–13 perform chorion gene amplification in the absence of genome-wide DNA replication. Because these follicle cells are eliminated after depositing chorion, intra-S phase checkpoint may not be necessary to maintain genomic stability. However, high-dose irradiation affected chorion production, which contributed to degeneration of defective embryos. Lastly, the egg chambers with various morphological defects that were able to survive apoptosis at mid-oogenesis most likely did not develop into functional embryos. It is interesting to note that the hatch rate of the eggs 2 days (19.3%) after irradiation was significantly higher than either day 1 or 3 (1.3% or 2.9%). It is likely that during late oogenesis the oocytes at certain developmental stages are more resistant to the harmful effect of high-dose irradiation and the precise stage remains to be studied.

In summary, we have performed a temporal analysis of the DNA damage response during oogenesis after high-dose irradiation and found a reduction of the fecundity and hatch rate of the eggs laid by irradiated females. The reduction of fecundity can be explained by the cell cycle arrest of the mitotically dividing cystoblast in germarium region 1, massive cell death in the germarium region 2, and delayed degeneration of egg chambers 7–10. The hatch rate of the eggs seems to be reduced because of the dorsal-ventral patterning defects, thin eggshell, and additional morphological defects of the germline cells. Most of the IR-induced defects during oogenesis were restored by 4 days post irradiation, suggesting that the germline stem cells were able to maintain the genomic stability and resume normal oogenesis after the defective germline cells were removed during oogenesis.
